# Effectiveness of management models for facilitating self-management and patient outcomes in adults with diabetes and chronic kidney disease

**DOI:** 10.1186/s13643-015-0072-9

**Published:** 2015-06-10

**Authors:** Edward Zimbudzi, Clement Lo, Marie Misso, Sanjeeva Ranasinha, Sophia Zoungas

**Affiliations:** Department of Nephrology, Monash Health, Clayton, Melbourne, VIC Australia; Monash Centre for Health Research and Implementation—MCHRI, School of Public Health and Preventive Medicine, Monash University, Clayton, Melbourne, VIC Australia; Diabetes and Vascular Medicine Unit, Monash Health, Clayton, Melbourne, VIC Australia; The George Institute for Global Health, University of Sydney, Sydney, NSW Australia; Level 1, 43-51 Kanooka Grove, Clayton, Melbourne, VIC 3168 Australia

**Keywords:** Diabetes, Chronic kidney disease, Self-management practices, Self-management models, Systematic review

## Abstract

**Background:**

Self-management models can be a very powerful resource in the health system provided they are well tailored to a particular disease and setting. Patient outcomes have been demonstrated to improve when self-management practices are embedded in the care of people with certain diseases. However, it remains unclear whether self-management models and specific components of these programmes can be implemented in order to effectively improve the care of people with diabetes and/or chronic kidney disease.

**Methods/Design:**

Medline (including Medline in-process), Excerpta medica database (EMBASE), Cumulative Index to Nursing and Allied Health (CINAHL) and all evidence-based medicine (EBM) will be systematically searched for randomised controlled studies comparing self-management models with usual care in patients with diabetes or chronic kidney disease. Two reviewers will independently assess articles for eligibility: extract data, evaluate risk of bias and complete quality assessment of included studies. The data will be tabulated and narratively synthesised. Meta-analyses will be performed if there is sufficient homogenous data.

**Discussion:**

This protocol utilises rigorous methodology as well as pre-specified eligibility criteria to comprehensively search for diabetes and kidney disease self-management models which have been compared with usual care in randomised controlled trials. The review is likely to provide insight into the effectiveness of current models for improving patient self-management, and this may address the key translational issue of how to integrate and tailor these self-management practices for patients with diabetes and chronic kidney disease.

**Systematic review registration:**

PROSPERO CRD42015017316.

**Electronic supplementary material:**

The online version of this article (doi:10.1186/s13643-015-0072-9) contains supplementary material, which is available to authorized users.

## Background

Chronic diseases are defined as illnesses that are prolonged in duration, do not often resolve spontaneously and are rarely cured completely [1]. They are the largest cause of death globally [2]. Among these diseases are diabetes and chronic kidney disease. The incidence and prevalence of diabetes mellitus has soared throughout the world, mainly due to the increase in type 2 diabetes, which in turn is largely related to the increase in overweight and obesity [3, 4]. It is projected that by 2025, there will be 380 million people with type 2 diabetes and 418 million people with impaired glucose tolerance [5]. Direct medical costs of treating diabetes and its complications during a lifetime are estimated to be $85,000 in the United States. In this regard, diabetes presents a huge financial challenge to the health system and the economy at large.

Chronic kidney disease can occur as a sequela of or independent of diabetes. Worldwide, CKD affects over 200 million people [6] and diabetes contributes 30–40 % of all end stage kidney disease (ESKD) cases [7]. In developed countries, diabetes-related kidney damage is the leading cause of treated end stage kidney disease accounting for approximately 50 % of cases [8]. Given the incidence of diabetes is increasing, a concurrent rising tide of people with kidney disease is anticipated.

Due to the complex nature of diabetes and CKD, it is not only important to prevent but to improve the entire continuum of care from prevention to treatment and self-management. Several self-management strategies have therefore been implemented to manage illnesses and minimise the impact on patients, families and the health system [9]. These strategies have been organised into models, which have produced some favourable outcomes including improvement of the physiological measures of disease, adherence to treatment, health service and self-reported health measures such as health-related quality of life [10]. However, the approach to self-management in these various chronic disease models has differed substantively. While some are centred on patient education, motivational interviewing and health coaching, others follow a much broader approach of the way the patient relates to health providers and the community.

Given the wide array of chronic disease health care models and self-management practices promulgated, it is possible to apply a model which poorly fits the particular chronic disease and setting of implementation. For example, often a “mismatch” between the needs of the patients and health care available exists due to the traditional acute care orientation of existing health systems [11–13]. Several studies have compared the outcomes of usual care (for various diseases in different settings) with one or other chronic disease health care models in order to identify the most effective means by which to provide care [13–15]. However, the effectiveness of these models in the management of people with diabetes and CKD has not been established.

A systematic review of the evidence is required to provide insight as to the most effective self-management models and the specific components of chronic disease health care models that can be implemented in order to improve the outcomes of people with diabetes and chronic kidney disease (CKD) [16, 17].

### Objectives of the systematic review

The objectives of this study are to investigate:

- The effectiveness of current diabetes and CKD management models in improving clinical outcomes of patients with diabetes and CKD,
- The common elements of a model of care that improves patient outcomes for diabetes and CKD and
- The effectiveness of current models of care in improving self-management in diabetes and CKD patients.

### Conceptual framework

**Fig. 1:**
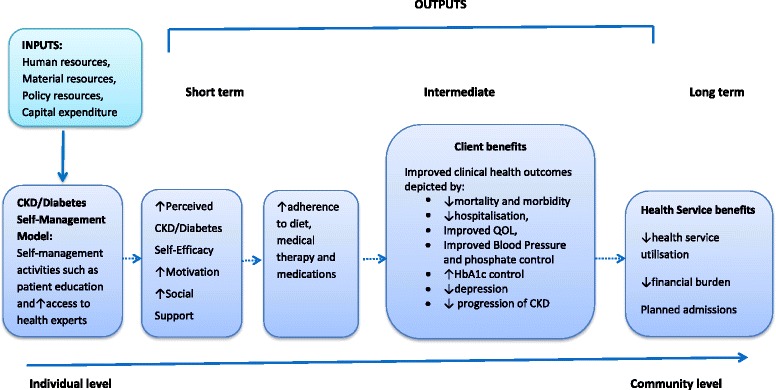
Programme logic model for a chronic kidney disease/diabetes self-management programme

As depicted in Fig. 1, a CKD/diabetes self-management model needs to have activities or interventions which are applied to the target population in addition to their usual care. These activities include patient education, patient reminders, motivational interviewing, health coaching, increased access to health experts and incentives. The impact of these interventions can be classified as short-term outcomes, intermediate outcomes and long-term outcomes.

Short-term outcomes measure the initial impact of an activity, for instance, improved self-efficacy. They capture the “potential” for continued change created through activities and their outputs. Intermediate outcomes are often few and are seen in individuals who continue to participate in self-management activities. They are the changes believed to be created by the project and not only impact individuals directly participating in the project’s activities but impact those connected to them such as families, friends and community partners. Long-term outcomes may be achieved after a lengthy duration (7–10 years), and they represent the ultimate goal for the project.

## Methods

### Systemic review design

A systematic review and meta-analysis which adopts methods outlined in the Cochrane Handbook for Systematic Reviews of Interventions guidelines [18] and conforms to the reporting guidelines of the Preferred Reporting Items for Systematic Reviews and Meta-Analyses for Protocols (PRISMA-P) statement recommendations [19] will be conducted. The methodology of this review will be guided by the PICOS format.

#### Population/participants

Adult patients (above 18 years) with diabetes and CKD in any healthcare setting (acute care, primary health care, family medical practice, general medical practice, clinics, outpatient departments, rehabilitation or community settings) in all countries.

#### Interventions

Chronic disease management models focusing on the healthcare provider or the patient will be considered. As a typical chronic disease management model has multiple interventions, relevant interventions will be classified into five groups [12]:

- Use of evidence-based planned care
- Reorganisation of practice systems and provider roles
- Improved patient self-management support
- Increased access to expertise
- Availability of clinical information

Relevant intervention components include [20]:

- Provider education—includes education materials or instructions given to the healthcare provider to aid with the management of a given chronic disease.
- Provider feedback—information given to healthcare providers regarding the care or results of care experienced by their patient.
- Provider reminders—prompts given to providers to perform specific patient care tasks.
- Patient education—materials and instructions given to patients to enhance the management of their chronic disease condition.
- Patient reminders—prompts given to patients to remind them to perform specific tasks related to the management of their disease condition.
- Patient financial incentives—payments (direct or indirect) to patients for achieving certain disease management goals.

Only studies whose chronic disease management models have included one or more of the above components will be eligible for inclusion.

#### Comparator

Usual or standard care must be clearly defined to be eligible for inclusion in this systematic review. This may be the chronic disease management programme that is already in place before a new model of care is introduced. Usual care will potentially present some challenges in this study since this may differ depending on setting.

#### Outcomes

##### Primary

Clinical indicators (*blood pressure*, eGFR and HbA1C): non-invasive measures of blood pressure performed by an automated machine or manually by a health practitioner will be accepted.

##### Secondary

Adherence to medical treatment: adherence to medication, diet, lifestyle changes or appointment keeping. All validated adherence measurement tools will be considered, including but not limited to direct observable behaviour, subjective self-reports (patient-reported outcome), objective monitoring of medication usage, objective physiological/biomedical measures, health outcomes or combined adherence measurements. Examples of some of the validated tools are the Medication Event Monitoring System (MEMS) [21], medication adherence report scale (MARS-5) [22], Morisky medication taking adherence scale (MMAS) [23] and the brief medication questionnaire (BMQ) [24].

Self-management behaviour: self-management (SM) can be defined as the “active management by individuals of their treatment, symptoms and lifestyle, physical and psychological consequences inherent with living with a chronic condition” [25]. To achieve adequate SM skills, individuals may require a series of SM interventions addressing their area of need. Effectiveness of SM models will be determined by evaluation of at least two key areas, such as, but not limited to whether people developed the skills to manage their own health and secondly, whether this has resulted in better health. Measures of impact of SM may include the patient activation measure (PAM) indicators such as patient knowledge, skill and confidence for SM and prediction of a range of behaviours including healthy behaviours, disease specific management behaviours and consumeristic type of behaviours [26]. The method of measurement of SM behaviour must be reported to be eligible for inclusion in this systematic review.

Health service utilisation: measures of the population’s use of the health care services available to them, incorporating economic indicators which are based on volume such as number of hospitalisations and number of visits per year. Ideally, chronic disease management models would aim for fewer hospitalisations due to the financial connotations associated with health service utilisation. It is very important to note that health service utilisation may be a long-term outcome and therefore may only be properly ascertained by studies with a reasonably longer follow-up period.

Health-related quality of life: only validated tools will be considered including EuroQol 5D [27], quality of life scale (QoLS) [28] and the kidney disease quality of life instrument (KDQOL) [29].

Adverse outcomes: adverse events such as hospitalisation and deaths will be considered in this review.

#### Study design/setting

Randomised controlled trials: For the purpose of this review, only randomised controlled studies and systematic reviews of randomised controlled studies, reporting adequate information to allow for estimation of at least one relevant outcome of the chronic disease management model as outlined above, will be considered.

The following *publication types will be excluded*: articles reporting non-randomised studies, narrative reviews, letters, editorials, commentaries, unpublished manuscripts, dissertations, government reports, books and book chapters, conference proceedings, meeting abstracts, lectures and addresses, and consensus development statements and guidelines.

Language: Studies published in English language will be included.

### Search methods

The following electronic databases will be used to identify relevant literature using a systematic search developed according to the selection criteria (Additional file 1):

- Medline
- Medline in-process and other non-indexed citations
- EMBASE
- CINAHL
- All Evidence-Based Medicine (EBM) Reviews incorporating The Cochrane Library, Cochrane Database of Systematic Reviews (Cochrane reviews), Database of Abstracts of Reviews of Effects (other reviews), Cochrane Central Register of Controlled Trials (clinical trials), Cochrane Database of Methodology Reviews (methods reviews), The Cochrane Methodology Register (methods studies), Health Technology Assessment Database (technology assessments), NHS Economic Evaluation Database (economic evaluations) and ACP Journal Club.

We will also search the bibliographies of relevant studies identified by the search strategy for identification of additional studies. The National Institute of Health Clinical Trials Register (https://clinicaltrials.gov/) and the Australian and New Zealand Clinical Trials Registry (https://www.anzctr.org.au/) will also be searched.

### Inclusion of studies

**Fig. 2:**
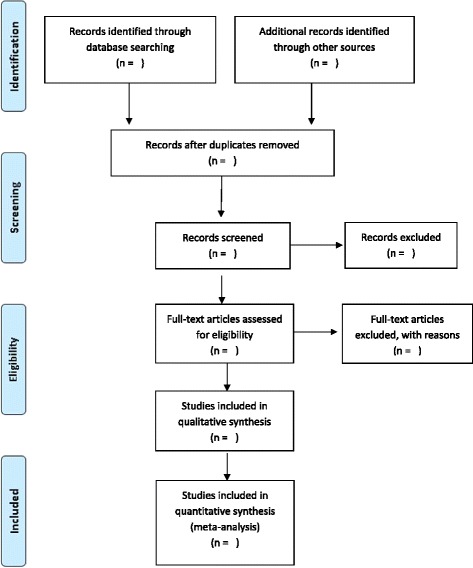
Prisma flow diagram showing how studies will be screened

To determine the literature to be assessed further, two reviewers (EZ and CL) will scan the titles, abstract sections and keywords of every record retrieved by the search strategy (Fig. 2). Full articles will be retrieved for further assessment if the information given suggests that the study meets the inclusion criteria. If there is any doubt regarding these criteria from the information given in the title and abstract, the full article will be retrieved for clarification. During the full text review, if the two reviewers are in doubt about the inclusion of any particular study, there will be an option of involving the third reviewer (MM). Level of agreement on study eligibility will be tested using the kappa statistic and 95 % confidence interval.

### Assessment of methodological quality

Methodological quality of the included studies will be assessed by two reviewers (EZ and CL) using the Monash Centre for Health Research and Implementation (MCHRI) template for appraisal of methodological quality of a randomised controlled trial (Additional file 2) [30]. This template uses a descriptive component approach to assess risk of bias as well as outline internal and external validity. Any disagreement will be resolved by discussion with MM to reach a consensus.

### Quality of evidence

The grading technique recommended by Guyatt and associates [28] will be used to assess the quality of the body of evidence for each outcome of interest. The effect estimate will be assessed for direction and size of the effect. In considering the quality of evidence for the effect, the following five factors will be considered: indirectness, risk of bias, imprecision, inconsistency and publication bias.

Overall quality will be classified as high, moderate, low and very low. Randomised controlled trials will start with high quality rating with each consideration being downgraded by 1 or 2 points as necessary. The final quality score will be interpreted as shown in Table 1.

**Table 1:** Grading the evidence (adapted from Guyatt et al. 2011[28])

Strength of evidence	Interpretation
High quality	Very confident that the true effect lies close to the estimate of the effect and therefore further research very unlikely to change our confidence in the estimate of effect
Moderate quality	Moderately confident in the effect estimate. The true effect is likely to be close to the estimate of the effect, but there is a possibility that it is substantially different. Further research likely to have an important impact on our confidence and may change the estimate
Low quality	Confident that the effect estimate is limited. The true effect may be substantially different from the estimate of the effect. Further research very likely to have an important impact on our confidence and is likely to change the estimate
Low	Very little confidence in the effect estimate. The true effect is likely to be substantially different from the estimate of effect

### Data extraction

Data will be extracted from included studies using a specially developed data extraction form according to the selection criteria. Information will be collected on general details (title, authors, reference/source, country, year of publication, setting), participants (age, sex, inclusion/exclusion criteria, withdrawals/losses to follow-up, subgroups), results (point estimates and measures of variability, frequency counts for dichotomous variables, number of participants, intention-to-treat analysis) and validity results.

### Data analysis and synthesis of evidence

Data will be presented in summary form and descriptively, in tables or narratively for each clinical question. Where appropriate, meta-analyses will be conducted.

Data will be summarised statistically if they are available, sufficiently similar and of sufficient quality. The Review Manager 5.3.5 software will be used for statistical analysis. Results will be expressed as relative risks (RR) with 95 % confidence interval (CI) for dichotomous outcomes and weighted mean differences (WMD) with 95 % CI for continuous outcomes. Results of clinically and statistically homogenous trials will be pooled to provide estimates of the effectiveness of the interventions. Clinical homogeneity will be satisfied when participants, interventions, outcome measures and timing of outcome measurement are considered to be similar. For trials that are clinically heterogeneous or present insufficient information for pooling, a descriptive analysis will be performed. Statistical homogeneity will be assessed using the *I*^2^ test where *I*^2^ values over 50 % indicate moderate to high heterogeneity [31]. Pooled results will be analysed using a random-effects model, assuming a degree of heterogeneity among self-management trials being sought here. Statistical significance will be set up at *P* ~ 0.05 for primary and secondary outcome measures.

#### Subgroup analysis

Subgroup analysis will be conducted according to age, gender and duration of intervention since these factors may cause variations in outcomes. The duration and type of self-management training will also be considered carefully.

A sensitivity analysis will be done according to risk of bias. For meta-analyses containing more than ten studies, funnel plots will be employed in order to investigate small study effects as well as publication bias [32]. Publication bias will be determined where a symmetrical inverted funnel plot indicates the absence of bias and an asymmetrical funnel plot indicates the presence of bias.

##### Narrative

A narrative synthesis will be performed using a framework that consists of the following four elements as highlighted by several authors [33–36]:

1. Developing a preliminary synthesis of findings of included studies.
2. Assessing the robustness of the synthesis which will involve performing a critical reflection with special emphasis to the methodology of the synthesis (focusing on the limitations and their possible impact on the results), evidence used (quality, reliability, validity and generalizability), assumptions made, discrepancies and uncertainties identified and how discrepancies were dealt with, areas where the evidence is weak or non-existent, possible areas for future research and, finally, a discussion of the evidence presented that will consider the “thick” and “thin” evidence and comment on similarities and/or differences between evidences.
3. Exploring relationships within and between studies will be done in three ways namely;
  i. *Moderator variables and subgroup analysis*—study characteristics that vary between studies or sample (subgroup) characteristics which might help explain differences in findings will be identified.
  ii. *Idea webbing and concept mapping*—idea webbing conceptualises and explores connections among the findings reported in the review studies and often take the form of a spider diagram.
  iii. *Qualitative case descriptions*—descriptions of outliers or exemplars of why particular results were found in the outcome studies.
4. Developing a theory of how the intervention works, why and for whom.

## Discussion

Our review utilises rigorous methodology as well as pre-specified eligibility criteria to comprehensively search for diabetes and CKD health care models and self-management practices which have been compared with usual care in randomised controlled trials. The search strategy for this review was developed in consultation with a methodological expert (MM). Furthermore, eligibility and risk of bias and extraction of data will be independently assessed by a team of two reviewers while a third reviewer will be available to adjudicate discrepancies.

This review may have some limitations. The study relies on published data so publication bias cannot be ruled out. We may also miss some relevant studies as we have limited the search to publication date and English language due to funding and time constraints.

Our review will provide insight into the effectiveness of current chronic disease health care models for improving patient self-management, and this may address the key translational issue of how to integrate and tailor these self-management practices to patients with diabetes and CKD. If the existing models are found to be less efficient, this review may flag avenues for further research.

## Additional files

Additional file 1:
**Search without RCT filter.** Search terms used to develop a comprehensive systematic search. Additional file 2:
**Template for critical appraisal of RCT.** Template for critical appraisal of randomised controlled trials.

## Abbreviations

CKD: chronic kidney disease
ESKD: end stage kidney disease
RRT: renal replacement therapy
CORE: The Centre for Outcomes Research Diabetes Model
HbA1c: glycated haemoglobin
eGFR: estimated glomerular filtration rate
MEMS: Medication Event Monitoring System
MARS-5: medication adherence report scale
MMAS: Morisky medication taking adherence scale
BMQ: brief medication questionnaire
SM: self-management
PAM: patient activation measure
QoLS: quality of life scale
KDQOL: kidney disease quality of life instrument
EuroQol: European quality of life scale
EMBASE: Excerpta medica database
CINAHL: Cumulative Index to Nursing and Allied Health
EBM: evidence-based medicine
MCHRI: Monash Centre for Health Research Innovation
WMD: weighted mean difference
CI: confidence interval
