# Supplementary material for: Effectiveness of management models for facilitating self-management and patient outcomes in adults with diabetes and chronic kidney disease
Source: Syst Rev. 2015 Jun 10;4:81. doi: 10.1186/s13643-015-0072-9 (PMC4489399; doi:10.1186/s13643-015-0072-9)
Supplement: Additional file 1: — Search without RCT filter. Search terms used to develop a comprehensive systematic search. [file 13643_2015_72_MOESM1_ESM.docx]

|  | **Additional file 1**  **Search without RCT filter** |
| --- | --- |
| 1 | exp Chronic Disease/ |
| 2 | (chronic adj3 (illness* or disease* or condition*)).mp. |
| 3 | chronic disease [therapy.mp](http://therapy.mp/). |
| 4 | or/1-3 |
| 5 | kidney diseases/ or anuria/ or diabetic nephropathies/ or hypertension, renal/ or hypertension, renovascular/ or renal insufficiency, chronic/ |
| 6 | chronic kidney [disease.mp](http://disease.mp/). |
| 7 | (chronic kidney or chronic renal).mp. |
| 8 | (CKD or CRD).mp. |
| 9 | diabetes mellitus/ or diabetes mellitus, type 1/ or wolfram syndrome/ or diabetes mellitus, type 2/ or diabetes mellitus, lipoatrophic/ or diabetic ketoacidosis/ or donohue syndrome/ |
| 10 | (MODY or NIDDM or T2DM or T2D).mp. |
| 11 | (non insulin* depend* or noninsulin* depend* or noninsulin?depend* or non insulin?depend*).mp. |
| 12 | ((typ? 2 or typ? II or typ?2 or typ?II) adj3 diabet*).mp. |
| 13 | (((late or adult* or matur* or slow or stabl*) adj3 onset) and diabet*).mp. |
| 14 | (IDDM or T1DM or T1D).mp. |
| 15 | (insulin* depend* or insulin?depend*).mp. |
| 16 | ((typ? 1 or typ? I or typ?1 or typ?I) adj3 diabet*).mp. |
| 17 | (insulin* defic* adj2 absolut*).mp. |
| 18 | or/5-17 |
| 19 | exp Consumer Participation/ |
| 20 | exp Self Care/ |
| 21 | exp Self Concept/ |
| 22 | ((self or self directed or self-directed or self monitor* or self-monitor* or symptom*) adj (care or help or manag* or efficacy or admin* or concept)).mp. |
| 23 | patient financial [incentives.mp](http://incentives.mp/). |
| 24 | health education/ or consumer health information/ or health literacy/ or patient education as topic/ |
| 25 | Health Communication/ |
| 26 | interdisciplinary communication/ |
| 27 | ((consumer or patient*) adj2 (educat* or information or particip* or behavio?r*)).mp. |
| 28 | ((health educat* or health information) adj2 (program* or intervention* or meeting* or session* or strategy* or workshop* or visit* or method* or material* orcampaign*)).mp. |
| 29 | access to [expertise.mp](http://expertise.mp/). |
| 30 | availability of clinical [information.mp](http://information.mp/). |
| 31 | Reminder Systems/ |
| 32 | patient [reminders.mp](http://reminders.mp/). |
| 33 | Pamphlets/ |
| 34 | (leaflet* or booklet* or poster* or pamphlet*).mp. |
| 35 | ((written or printed or oral) adj information).mp. |
| 36 | (provider adj2 (educat* or feedback or remind* or behavio?r)).mp. |
| 37 | Health Care Reform/ |
| 38 | health care [reform.mp](http://reform.mp/). |
| 39 | exp Patient Care Management/ |
| 40 | (care co-ordinat* or care coordinat*).mp. |
| 41 | chronic disease management [model.mp](http://model.mp/). |
| 42 | exp "Continuity of Patient Care"/ |
| 43 | continuity of patient [care.mp](http://care.mp/). |
| 44 | behavio?r [change.mp](http://change.mp/). |
| 45 | models, nursing/ or models, organizational/ |
| 46 | or/19-45 |
| 47 | (model* or strateg* or intervention* or program*).mp. |
| 48 | 22 or 27 or 28 or 36 or 40 or 43 or 44 |
| 49 | 47 and 48 |
| 50 51 | 46 or 49  4 and 18 and 50 |

Note: Validated filters for identifying randomised controlled trials and systematic reviews will be used. Search will be used in Ovid for all databases except for Cinahl for which this string will be translated. The search will be limited to articles from 1994 as it is deemed that relevant studies could have been reported in the past 20 years.
