# Supplementary material for: Effectiveness of management models for facilitating self-management and patient outcomes in adults with diabetes and chronic kidney disease
Source: Syst Rev. 2015 Jun 10;4:81. doi: 10.1186/s13643-015-0072-9 (PMC4489399; doi:10.1186/s13643-015-0072-9)
Supplement: Additional file 2: — Template for critical appraisal of RCT. Template for critical appraisal of randomised controlled trials. [file 13643_2015_72_MOESM2_ESM.doc]

**Additional file 2 Template for critical appraisal of a randomised controlled trial**

Document evidence from the article in quotation marks.

| **Study ID** | |  | | |
| --- | --- | --- | --- | --- |
| **Study citation** | |  | | |
| **EXTERNAL VALIDITY – IS THIS STUDY AND ITS RESULTS GENERALIZABLE TO MY SYSTEMATIC REVIEW QUESTION?** | | | | |
| **Patient/population/ participants** | | | Describe whether they were gender specific, had a particular condition or the general population, age and any other relevant characteristics (e.g. BMI) | |
| **N** | | | Where possible, list the number of participants that were:   - Screened - Enrolled - Allocated/randomised - Assessed - Followed up | |
| **Setting** | | | List where the intervention was conducted and assessed ie. hospital, clinic, community and/or university setting. | |
| **Intervention/indicator** | | | Describe the intervention in as much detail as possible e.g. medication type, dose, duration, intervals. | |
| **Comparison/control** | | | Describe the comparison in as much detail as possible e.g. medication type, dose, duration, intervals. | |
| **Outcomes** | | | List what the study measured (e.g. weight, BMI, HbA1c) as primary outcomes and secondary outcomes. If the outcomes are not relevant to your systematic review, list these as measured but not relevant to your systematic review. | |
| **Inclusion Criteria** | | | Yes  No  Not reported |  |
| **Exclusion Criteria** | | | Yes  No  Not reported |  |
| **Does the study have a clearly focused question and/or PICO?** | | | Yes  Partial  No  Not reported | Consider if the question is ‘focused’ in terms of:  – the population studied  – the intervention given or exposure  - the comparison(s)  – the outcomes considered |
| **Does the study have specified inclusion/exclusion criteria?** | | | Yes  Partial  No | Consider if the inclusion or exclusion of patients was clearly defined a priori. |
| **If there were specified inclusion/ exclusion criteria, were these appropriate?** | | | Yes  Partial  No  N/A | Consider if:  - the eligibility criteria used to specify the patients, interventions/ exposures and outcomes of interest. |
| **Were the outcomes measured appropriate?** | | | Yes  Partial  No  Not reported | Consider if the outcomes measured are appropriate and important outcome. |
| **Was there sufficient duration of follow-up?** | | | Yes  Partial  No  Not reported | May need to check with clinicians regarding what is sufficient duration for important events to occur. |
| **INTERNAL VALIDITY – HAS THIS STUDY BEEN CONDUCTED RIGOROUSLY IN ORDER TO REDUCE BIAS?** | | | | |
| **SELECTION BIAS** | **Did the study have an adequate method of randomisation?** | | Yes  No  Not reported | Method of randomisation is considered adequate when patient’s allocation is entirely due to chance.  Adequate methods include:  - computer-generated random numbers  - table of random numbers  - coin tossing  Inadequate methods include:  - systematic methods (DOB, case record number, day of the week presenting)  - sequence may be related to confounding variable  - allows foreknowledge of assignment. (These studies should therefore be classed as Controlled Clinical Trials rather than RCTs.) |
|  | **Was allocation to intervention group concealed?** | | Yes  No  Not reported | Concealment of allocation is considered adequate when the person responsible for allocation cannot influence which group a patient is randomised to.  Adequate methods of concealment of randomisation include:  - Centralised or pharmacy-controlled randomisation  - On-site computer based system with a randomisation sequence that is not readable until allocation  - Other approaches with robust methods to prevent foreknowledge of the allocation sequence to clinicians and patients  Inadequate approaches to concealment of randomisation  - Open random numbers lists  - Serially numbered envelopes (even sealed opaque envelopes can be subject to manipulation) |
| **PERFORMANCE BIAS** | **Were patients blind to intervention group?** | | Yes  No  Not reported | Consider:  - how the study has attempted to maintain blinding  - if there is any indication that patients were aware of intervention group  - the fact that blinding is not always possible  - if every effort was made to achieve blinding |
|  | **Were investigators and care providers blind to intervention group?** | | Yes  Partial  No  Not reported | Consider:  - how the study has attempted to maintain blinding  - if there is any indication that investigators or care providers were aware of intervention group  - the fact that blinding is not always possible  - if every effort was made to achieve blinding |
|  | **Aside from the experimental intervention, were the groups treated the same?** | | Yes  Partial  No  Not reported | To be sure it’s the intervention which is responsible for the effect. |
| **DETECTION BIAS** | **Were outcome assessors blind to intervention group?** | | Yes  Partial  No  Not reported | Consider:  - If the outcome is objective (e.g. death) then blinding is less critical.  - If the outcome is subjective (e.g. symptoms or function) then blinding of the outcome assessor is critical. |
|  | **Were all outcomes measured in a standard, valid and reliable way?** | | Yes  Partial  No  Not reported | Where outcome measures require any degree of subjectivity, some evidence should be provided that the measures used are reliable and have been validated prior to their use in the study. |
|  | **Were outcomes assessed objectively and independently?** | | Yes  Partial  No  Not reported | Independence of assessment is important where the result of one outcome may effect the interpretation of another.  When outcomes are objectively assessed, their independence from each other is less important. |
| **ATTRITION BIAS** | **What percentage of the individuals recruited into each arm of the study dropped out?** | | X% treatment  X% control/ comparison | Consider:  - if all patients who entered the trial were properly accounted for and attributed at its conclusion.  - why patients dropped out, as well as how many.  - the drop out rate may be expected to be higher in studies conducted over a long period of time. |
|  | **Were all the subjects analysed in the groups to which they were randomly allocated (ie intention to treat analysis)?** | | Yes  No  Not reported | Consider:  - if analysis was as per protocol or intention to treat  - number of crossovers  - reason for crossover |
| **REPORT BIAS** | **Is the paper free of selective outcome reporting?** | | Yes  Partial  No  Not reported | Consider:  - if all the planned outcomes were measured  - if all the measured outcomes were reported  - if any additional or composite outcomes were measured.  This is difficult to determine if there isn’t a protocol. |
| **CONFOUNDING** | **Were the groups similar at baseline with regards to key prognostic variables?** | | Yes  Partial  No  Not reported | Key prognostic variable include age, sex, disease severity. If the randomisation process worked (that is, achieved comparable groups) the groups should be similar, however particularly in small studies, some variations are very likely.  There should be some indication of whether differences between groups are clinically important. May need to check with clinician for this information. |
|  | **If confounding was present, was it controlled for?** | | Yes  Partial  No  Not reported | Consider if any effort was made to control for confounding – whether the participants were exposed to other factors that may lead to an effect similar to that expected as a result of the intervention ie. for an exercise study, was one group more motivated than the other? which may lead to higher intervention effect. Look for comments about stratifying for ages etc. |
| **OTHER INTERNAL VALIDITY/BIAS** | **Were there any conflicts of interest in the writing or funding of this study?** | | Yes  No  Not reported | Consider:  - if any of the authors are/were employed, sponsored etc by pharmaceutical companies, or have other financial/other ties  - if any commercial companies were involved in funding, writing, editing, data analysis or manuscript approval |
|  | **Was the study sufficiently powered to detect any differences between the groups?** | | Yes  Partial  No  Not reported | Consider:  - if an adequate sample size calculation was undertaken  - if the required sample size recruited and retained  - for which outcomes the study was powered  - if confidence intervals include a clinically important difference, the study was underpowered  NB this is less important if significant differences were found. |
|  | **For cross over studies - was the washout period adequate?** | | Yes  No  Not reported  NA | Consider:  - the likely duration of action of the treatment being tested. |
|  | **If statistical analysis was undertaken, was this appropriate?** | | Yes  Partial  No  Not reported  N/A | Consider:  - whether the authors performed any statistical tests or just presented figures  - if the statistical analysis was planned a priori  - if the data were analysed accordingly to the study protocol.  - the type of data and the statistical tests used. (Please refer to the CCE workbook as required)  - use of parametric versus non-parametric tests; whether the data has been checked for normality  - if the tests used are obscure, why did the authors used them and have they included a reference.  - if point estimates and measures of variability were presented for the primary outcome  - if subgroups were analysed appropriately  - if potential confounders were identified and taken into account in the analysis  - if there was any adjustment made for multiple testing  - if missing data was handled appropriately |
| **Comments** | | | *Add any other relevant comments, including if this is likely to influence the results of the study* | |
| **What is the overall risk of bias?** | | | Low  Moderate  High  Insufficient information | *Low* ***-*** *All of the criteria have been fulfilled or where criteria have not been fulfilled it is very unlikely the conclusions of the study would be affected.*  *Moderate - Some of the criteria have been fulfilled and those criteria that have not been fulfilled may affect the conclusions of the study.*  *High - Few or no criteria fulfilled or the conclusions of the study are likely or very likely to be affected.*  *Insufficient information – not enough information provided on methodological quality to be able to determine risk of bias.* |

**Cited in full as:** Monash Centre for Health Research and Implementation (MCHRI) Evidence Synthesis Program template for critical appraisal of a randomised controlled trial (2013), MCHRI – Monash University and Monash Health, Melbourne, Australia (*adapted from* Critical Appraisal Templates (2010) Centre for Clinical Effectiveness, Southern Health, Melbourne, Australia).
